# Allochrony in Atlantic Lumpfish: Genomic and Otolith Shape Divergence Between Spring and Autumn Spawners

**DOI:** 10.1002/ece3.70946

**Published:** 2025-02-14

**Authors:** Mathilde Horaud, Núria Raventós, Kim Præbel, Carles Galià‐Camps, Cinta Pegueroles, Carlos Carreras, Marta Pascual, Victor M. Tuset, Shripathi Bhat, Arve Lynghammar

**Affiliations:** ^1^ Faculty of Biosciences, Fisheries and Economics, The Norwegian College of Fishery Science UiT The Arctic University of Norway Tromsø Norway; ^2^ Centre d'Estudis Avançats de Blanes (CEAB‐CSIC) Blanes Spain; ^3^ Otolith Research Lab Centre d'Estudis Avançats de Blanes (OTOLAB‐CEAB‐CSIC) Blanes Spain; ^4^ Inland Norway University of Applied Science Elverum Norway; ^5^ Department of Genetics, Microbiology and Statistics University of Barcelona (UB) Barcelona Spain; ^6^ Institute for Research on Biodiversity (IRBio) University of Barcelona (UB) Barcelona Spain; ^7^ Department of Genetics and Microbiology Universitat Autònoma de Barcelona Barcelona Spain; ^8^ Unidad Asociada ULPGC‐CSIC, Instituto de Oceanografía y Cambio Global, IOCAG Universidad de las Palmas de Gran Canaria Telde Gran Canaria, Canary Islands Spain

**Keywords:** allochrony, atlantique lumpfish, otolith shape, spawning grounds, whole genome sequencing

## Abstract

Allochrony is a form of reproductive isolation characterized by differences in the timing of spawning and may play a crucial role in the genetic and phenotypic divergence within species. The Atlantic lumpfish ( *Cyclopterus lumpus* ) is known to spawn in spring and autumn. However, the role of allochrony on the genomic structure of this species has not been addressed. Here, by combining whole genome sequencing data and otolith shape of 64 specimens, we explore the evolutionary drivers of divergence in Atlantic lumpfish, focusing on spring and autumn spawners sampled at two well‐separated spawning grounds along the Norwegian coast. Overall, we identified pronounced genomic and morphologic differences between the two spawning groups. Genomic differences between the two groups were concentrated in three chromosomes, with a region of chromosome 1 encompassing the same single nucleotide polymorphisms (SNPs) driving differential season spawning for both localities, suggesting parallel responses. The functional analysis of the SNPs in this region revealed genes associated with responses to environmental stressors, possibly adaptations to seasonal variations at high latitudes. The morphological analysis of otoliths supported these findings, showing differences compatible with adaptations to seasonal light availability. The presence of genomic islands of divergence, alongside a general lack of differentiation across the mitochondrial genome, suggest recent and rapid selection processes potentially modulated by ongoing gene flow. This study underscores the importance of considering temporal genetic structures, particularly for species with bimodal spawning time, in conservation and management strategies to prevent overexploitation and optimize breeding programs.

## 1 Introduction

Allochrony, or differences in spawning time, significantly shapes intra‐specific genetic and phenotypic divergence by inducing variations in the timing of key life history events, such as spawning. This phenomenon increases a genetic structure by restricting gene flow and promoting assortative mating, potentially leading to reproductive isolation and sympatric speciation (Taylor and Friesen 2017). Advanced genomic analyses are crucial for understanding these complex genetic variations and their temporal dynamics. Recent technological advances in sequencing and bioinformatics have enhanced our ability to generate and analyze large‐scale genomic data, essential for comprehensive population genomics studies (Formenti et al. 2022; Nigenda‐Morales et al. 2023; Pegueroles, Pascual, and Carreras 2023). Whole genome sequencing of even a small number of individuals allows us to assess genetic differentiation among populations and identify candidate regions of adaptation (da Fonseca et al. 2024; Galià‐Camps et al. 2024; Knutsen et al. 2022; Kurland et al. 2024). Furthermore, allochrony influences ecological and morphological diversity by exposing individuals to different environmental conditions. This can result in the emergence of distinct ecotypes and morphotypes, each characterized by unique life history traits (Santos et al. 2011; Bitz‐Thorsen et al. 2020). In teleosts, otolith analyses have proven effective in studying these divergences, revealing variations in habitat use, hatching times, and even aiding in the delineation of populations or cryptic species (Han, Iizuka, and Tzeng 2009; Heim‐Ballew et al. 2024; Sadighzadeh et al. 2012; Tuset et al. 2019). Variations in the timing of spawning have been observed in different species of marine fish where divergence at some loci have been described associated to the different behavior (Callihan et al. 2008; Hearsey and Kinziger 2015; O'Malley, Camara, and Banks 2007; van Damme et al. 2009). The Atlantic lumpfish ( *Cyclopterus lumpus* , Linnaeus 1758) is a suitable example for exploring the effects of allochrony on genetic structure and phenotype. Spawning is characterized by an extended season going from early spring to late autumn with fidelity to both spawning time and site over years (Kennedy 2018; Kennedy et al. 2015; Kennedy and Ólafsson 2019). With its broad distribution in the North Atlantic and adjacent Arctic regions (Mecklenburg et al. 2018), Atlantic lumpfish migrates from offshore feeding grounds to shallow coastal waters to spawn. The spawning peak in spring coincides with intensified fishing pressure (Lovdata 2023) on mature females, which are harvested for their roe, primarily a delicacy for human consumption, and to a lesser extent as broodstock for artificial cleaner fish hatching (Powell, Scolamacchia, and Garcia de Leaniz 2018).

In aquaculture, particularly in Norway and the United Kingdom, lumpfish play a crucial role in controlling sea lice ectoparasites in salmon farms (Powell, Scolamacchia, and Garcia de Leaniz 2018). While in autumn the fishery is restricted by law, only few Norwegian vessels get a permit to fish on autumn spawners (Directorate of Fisheries 2023). Spring and autumn spawners differ not only in fishing pressure but also in the environmental conditions they are facing.

Seasonal variations in biotic and abiotic conditions along the Norwegian coast are markedly distinct between spring and autumn (Ibrahim et al. 2014). For example, coastal temperatures reach their peak after spring bloom, aligning with a reduction in nitrate concentrations that typically occurs during the spring bloom. Conversely, winter is characterized by more turbulent mixed layers, exhibiting lower temperatures and elevated nitrate levels (Ibrahim et al. 2014). Additionally, light conditions in this region of the globe also undergo significant changes; the spring season experiences the midnight sun, providing continuous daylight, whereas autumn transitions towards the polar night, resulting in substantially reduced sunlight availability.

The variation in spawning times, coupled with differences in fishing pressures and environmental conditions, raises critical management questions regarding the Atlantic lumpfish. A key inquiry is whether the spring and autumn spawners constitute distinct stocks requiring separate management strategies. Understanding the stock structure of the Atlantic lumpfish is important, as it is crucial for setting effective regulations that promote sustainable fishing practices and resource conservation. Accurate stock assessments are essential for preventing overexploitation and ensuring the long‐term sustainability of fisheries (Hilborn et al. 2020). For lumpfish, which exhibit supposed philopatric behavior (Kennedy et al. 2015; Kennedy and Ólafsson 2019) but could form mixed foraging aggregates, sampling mature individuals at the spawning sites is essential to identify the spawning stocks. This approach has been fundamental in other philopatric species to help in accurately determining their genetic structure and unveiling the origin of individuals captured in the sea (Clusa et al. 2018).

The present study aims to improve our understanding of how allochrony influences population structure and adaptation in the Atlantic lumpfish. Using morphological approaches and advanced genomic analyses, we investigate the impact of timing of spawning on otolith's morphology, population structure, and potentially adaptive evolution. Our objectives are threefold: (i) to identify and quantify otolith shape differences between the spring and autumn spawning groups, assessing potential selection acting on these traits; (ii) to explore the genetic diversity and divergence at the nuclear level, to understand the adaptive significance of genomic regions exhibiting divergence and focusing on the characterization of parallel genetic changes; and (iii) to examine the mitochondrial lineage of each potential spawning group, aiming to determine if mitochondrial DNA patterns align with any nuclear divergence.

The findings of this study will provide crucial insights for both evolutionary biology and fisheries management, potentially revealing previously unrecognized stock structure and its potential impact on aquaculture in this commercially important species.

## 2 Materials and Methods

### 2.1 Sampling

**FIGURE 1:**
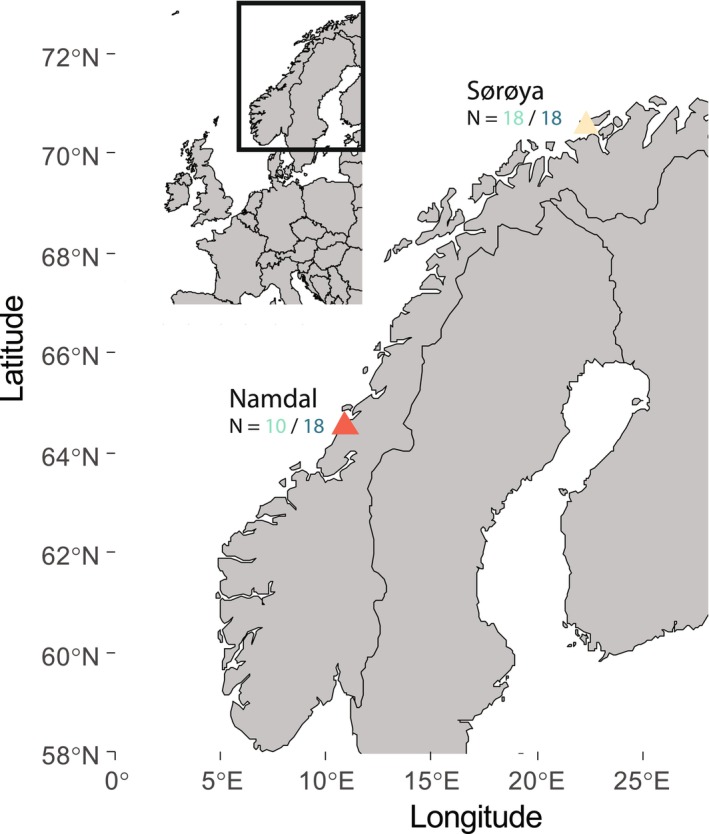
Origin of samples. Sampling sites (triangles) at the Norwegian coast with a number of mature females sampled from spring (light green) and autumn (dark blue).

Two distinct spawning grounds along the Norwegian coast, Namdal (NAM) and Sørøya (SOR), separated by more than 800 km, could be sampled for mature female lumpfish in spring and autumn (Figure 1, Table 1). A total of 64 individuals were captured using gillnets (Table 1). The collection of lumpfish was facilitated by two cleaner fish producer companies, Sørøya Rensefisk AS and Namdal Rensefisk AS. Only few cleaner fish producer companies are allowed to fish for mature females outside of the spring spawning season, which significantly restricts access to autumn spawner samples. In consequence, only three vessels were operating in autumn 2021 (Open data: catch data Directorate of Fisheries, 2024), thus limiting the number of sampling localities for this season. The total length of each lumpfish was measured (Table S1). A fin clip about 1 cm^2^ was taken for subsequent DNA analysis, preserved in 96% ethanol and stored at −20°C. Additionally, the left and right sagittal otoliths were removed from each specimen using the “medial cut” method as described by Albert et al. (2002), cleaned in distilled water, and stored dry in numbered airtight bags (Ziploc(R), Johnson & Son Inc).

**TABLE 1:** Sampling location and number of mature females (*N*) per season and year analyzed with whole genome sequencing.

Location (acronym)	Latitude and longitude	Sampling season	Year	*N*
Sørøya (SOR)	70°39′42.7″ N 22°00′00.6″ E	Spring	2021	8
		Autumn	2021	18
		Spring	2022	10
Namdal (NAM)	64°30′36.6″ N 10°46′14.7″ E	Spring	2022	10
		Autumn	2021	18

### 2.2 Spring‐Autumn Spawner Phenotypic Analyses

Otoliths processing was performed at CEAB's Otolith Research Lab (https://www.ceab.csic.es/en/external‐services/otolith‐analysis/). For age estimation, the left otolith of each fish was chosen and mounted face down on a microscope slide using thermoplastic glue (Crystalbond 509). The right otolith was used similarly when the left otolith was not of good quality. Readings were carried out under reflected light using a Motic SMZ 171 stereomicroscope connected to a Euromex camera and its associated image analysis software ImageFocusAlfa. To better expose the annual increments, otoliths were slightly hand polished along the sagittal plane using different grained sandpapers from 40 μm to 1 μm lapping film, (3M Imperial); the hyaline (winter growth) zones including the margin were counted to estimate the age of each individual (Table S1). To test if there were significant differences in age distribution between spring and autumn, a two‐sample Kolmogorov–Smirnov test was performed in R (R Core Team 2023). Similarly, significant differences in total length between spring and autumn spawners were tested in both localities using the same test.

For the morphological analysis, unpolished right otoliths, which were not used for age estimation, were employed. This resulted in a total of 21 otoliths from spring spawners and 20 from autumn spawners. To ensure accurate measurements and reduce distortion errors during the normalization process (Tuset, Lombarte, and Assis 2008), the otoliths were oriented with the inner side (*sulcus acusticus*) facing up and the *rostrum* to the left on the horizontal plane. The digitization of the otoliths was performed using the same setup that was used for age estimation (see above). The analyses of the otolith contour were based on wavelet functions (Parisi‐Baradad et al. 2005, 2010). From each contour, a total of 512 Cartesian coordinates on each orthogonal projection of the otolith were extracted using the wavelet representation routines developed in MATLAB language and implemented in the AFORO v‐1.1.2 (‘Anàlisi de FORmes d'Otòlits’) web page (http://aforo.cmima.csic.es/index.jsp) (Lombarte et al. 2006). The 4th (medium‐small detail) wavelet was selected for further analyses, as it is recommended to capture the optimal level of detail for identification of intra‐specific populations or morphotypes (Sadighzadeh et al. 2014; Abaad et al. 2016; Tuset et al. 2019). A principal component analysis (PCA) was performed in R using pcrcomp function from stats v‐4.3.1 package (R Core Team 2023) to reduce the wavelet functions with no loss of information on the otolith shape (Sadighzadeh et al. 2012; Tuset et al. 2019, 2021). Only the PCA variables that explained more than 1% of variability were used for subsequent analyses. Among the non‐parametric classification methods, the artificial neural network (ANN) was chosen for phenotype comparison across different spawning groups due to its high accuracy and frequent application in otolith studies, such as fish classification (El Habouz et al. 2016; Tuset et al. 2021), aging (Robertson and Morison 1999; Moen et al. 2018), and microchemistry (Hanson et al. 2004; Mercier et al. 2011). This classifier operates on a network architecture consisting of neurons, with three main layers: the input layer (morphological variables), multiple hidden layers (nodes from *i* = 1…*n*), and the output layer (spawning groups). A multi‐layer perceptron (MLP) architecture and a back‐propagation gradient algorithm were utilized for calibration (El Habouz et al. 2016; Ciaburro and Venkateswaran 2017). The Leave‐One‐Out Cross‐Validation (LOOCV) method was implemented (Marti‐Puig et al. 2016, 2020). In this approach, each otolith was used once as the validation data while the remaining otoliths constituted the training set. This process was repeated until each otolith was used for validation with 1000 repetitions for each analysis. The classifications were conducted using the R packages caret v‐6.0.94 (Kuhn 2008) and RSNNS (Bergmeir and Benítez Sánchez 2012), with optimal hyperparameters (hidden units) determined during preliminary tuning. The performance of the ANN model was evaluated using a confusion matrix, which compared the predicted spawning season against the actual season for each otolith. From the confusion matrix, various metrics such as accuracy and the Kappa statistic were calculated. These metrics provided insights into the model's classification capabilities. Finally, to identify which principal component in the ANN model had a higher influence on the shape variation between spring and autumn spawners, a variable importance analysis was conducted using the varImp function in caret package (Kuhn 2008) in R.

### 2.3 DNA Extraction, Library Preparation, and Whole Genome Re‐Sequencing

DNA was extracted from the individual fin clips using DNeasy Blood & Tissue Kit (Qiagen, Hilden, Germany), following the manufacturer's protocol. The DNA integrity of the extracts was checked using 1% agarose gel electrophoresis. The concentration of double‐stranded DNA was assessed using the Quant‐iT PicoGreen dsDNA Assay Kit (Invitrogen, Thermo Fisher Scientific, Oregon, US). All DNA extracts were kept at −18°C until shipping to NOVOGENE (UK) Co. (Cambridge, United Kingdom), for sequencing on an Illumina NovaSeq 6000 sequencing platform (Illumina Inc., CA, US). DNA purification, library preparation, and sequencing using 150 base‐pair (bp) paired‐end (PE) chemistry were performed by NOVOGENE. Each individual was sequenced to a minimum output of 9 Gb aiming for a mean coverage of *ca*. 15×, considering that the reference genome size has 572.9 Mb (NCBI RefSeq Accession GCA_009769545.1; Holborn et al. 2022). Two different library preparation protocols were used depending on the DNA concentration of each sample. For samples passing the quality check, the automatized library preparation protocol from NOVOGENE was used. For samples with an insufficient DNA concentration, the low input DNA library preparation was handled manually by adjusting all volumes (Table S2).

### 2.4 Sequence Mapping, Variant Calling, Single Nucleotide Polymorphism (SNP) Filtering, and Genetic Diversity

Forward and reverse sequence reads from each sample were merged and quality checked using FASTQC v‐0.11.9 (Andrews 2010) and MULTIQC v‐1.14 (Ewels et al. 2016), before and after removing adaptors and low‐quality sequences using default parameters in TRIMMOMATIC v‐0.39 (Bolger et al. 2014). Clean reads were mapped to the available Atlantic lumpfish reference genome (GCA_009769545.1; Holborn et al. 2022) with BWA‐MEM v‐2.2.1 (Vasimuddin et al. 2019) using default parameters. After the initial mapping, we sorted the bam file with SAMtools v‐1.17 (Danecek et al. 2021) by chromosomes. Picard MarkDuplicates (Picard Toolkit. Broad Institute, GitHub repository, http://broadinstitute.github.io/picard/) was used with default parameters to flag and remove PCR duplicates. SNPs were called using BCFtools v‐1.18 (Li 2011). SNPs were filtered with VCFtools v‐0.1.16 (Danecek et al. 2011) keeping only genotypes with a read depth equal or greater than 5 (–minDP 5), and sites with a Minor Allele Frequency (MAF) greater than or equal to 0.02 (–maf 0.02) were included. Only biallelic SNPs were kept (–min‐alleles 2 and –max‐alleles 2), and indels were removed (–remove‐indels). Finally, only genotypes being present in at least 90% of the individuals (–max‐missing 0.9) were kept. Observed heterozygosity (*H*_o_) of each sample was calculated using VCFtools (–het). Using the same program, the nucleotide diversity (*π*) was calculated on a per‐site basis using non‐overlapping windows of 10,000 bp (–window‐pi). To test if *H*_o_ and *π* differs between spring and autumn spawners, two separate *t*‐tests were performed in R using *t*‐test function in the stats package. Normality of the data was checked with the Shapiro–Wilk test function in stats v‐3.6.2 package in R and visualized by using the ggdensity function from ggpubr v‐0.6.0 package (Kassambara 2023).

### 2.5 Nuclear Genetic Differentiation

Two analyses of molecular variance (AMOVA) using the poppr package (version 2.9.5; Kamvar, Tabima, and Grünwald 2014) in R were conducted. The first analysis assessed genetic differentiation among Atlantic lumpfish across two localities and between seasons within each locality. The second analysis evaluated genetic differentiation between seasons and among localities within each season. This statistical approach allowed us to quantify and compare the variance explained by each factor (i.e., localities and seasons) to determine which contributes more to genetic variation.

To visualize the genetic differentiation across space and time, independent multidimensional scaling (MDS) analyses were conducted for each sampling site based on pairwise identity‐by‐state (IBS) distances using the plink v‐1.9b_6.13 software (Purcell et al. 2007). The results were visualized using the ggplot2 v‐3.4.3 package (Wickham, Chang, and Wickham 2016) in R.

To elucidate whether the observed genetic differentiation between spring and autumn spawners is homogeneous across the genome, we calculated *F*_ST_ estimates using Weir and Cockerham's (1984) method with VCFtools (–weir‐fst‐pop), using non‐overlapping windows of 10,000 bp (–fst‐window‐size). The *F*_ST_ estimates were then visualized as Manhattan plots using the R package CM_PLOT v‐4.4.3 (Yin et al. 2021), presenting a comprehensive snapshot of the genetic divergence patterns between spring and autumn samples in the two locations across the genome. Windows exceeding the 99.9th percentile of the empirical distribution of *F*_ST_ values in both localities were considered for further analysis as areas of high differentiation (Lopes et al. 2016; Marcos et al. 2022; Wilkinson et al. 2013). Their significance was tested using Rosner's test (Rosner 1983) with the EnvStats package in R (Millard 2013) and with a significance level of 0.01. To check that the divergence observed is really linked to strong genetic differentiation between spring and autumn spawners, we also computed and plotted for each SNP the absolute allele frequency difference between the two spawning groups in each localities using the VCFtools (–freq) and R package CM_PLOT v‐4.4.3. A zoom in on chromosome 1 was carried out with both *F*_ST_ and allele frequency differences between spring and autumn spawners since this chromosome is the only one with regions significantly separating the two groups in both locations (see Section 3). Outlier loci were identified by calculating individual SNP *F*_ST_ values. Similar to the previous analysis, the SNPs exceeding the 99.9th percentile in both of our comparisons (SOR: spring vs. autumn and NAM: spring vs. autumn), and that were significantly different according to Rosner's test, were considered as putatively outlier SNPs. The presence of potential chromosomal inversion on this chromosome was tested using the methodology developed by Galià‐Camps et al. (2024) with sliding windows of 1000 bp and a 250 bp step.

### 2.6 Functional Analysis of Genomic Regions of Interest

We characterized the genomic category (genic or intergenic) of the identified outlier SNPs based on a genome annotation file (GFF) containing only the longest isoform per gene that we created based on the GFF of the reference genome. We used BEDTools v2.30.0 (Quinlan and Hall 2010) to identify which SNPs corresponded to genic regions and subsequently extracted the Gene Ontology (GO) Terms associated with the coding sequences with eggNOG‐mapper v‐2.1.9 (Huerta‐Cepas et al. 2017). GO terms were summarized and aggregated with the REVIGO platform (Supek et al. 2011). To classify the SNPs in genic regions according to their location in exons, introns, or regulatory regions, we used the website IGV web app (Thorvaldsdóttir, Robinson, and Mesirov 2013). For each gene with a SNP in an exon region, the coding sequences (CDS) of this gene were extracted based on the GFF file. The CDS were then translated into amino acids using Geneious Prime 2023.2.1 (https://www.geneious.com). If the polymorphism resulted in an amino acid change, the SNP was identified as non‐synonymous (nsSNPs). The properties of the amino acids coded by the nsSNPS were assessed based on R‐group (basic, acidic, polar, and non‐polar), nutrition characteristics (essential and non‐essential), and metabolic fate (glucogenic and ketogenic) and were obtained from https://microbenotes.com/amino‐acids‐properties‐structure‐classification‐and‐functions/#properties‐of‐amino‐acids (accessed August 2024). The genotypes of each individual identified as spring or autumn spawners at the nsSNPs were kept with VCFtools. For each sampling season, combining the individuals of both localities, a chi‐square test was conducted to assess whether each locus deviated from Hardy–Weinberg equilibrium. We also evaluated if the genotype frequencies between spring and autumn spawners for each locus were significantly different with a chi‐square test, also combining the individuals of both localities.

### 2.7 Mitogenomes' Assembly and Analysis

The mitogenomes were assembled for all 64 individuals using NOVOPlasty v‐4.3.1 (Dierckxsens, Mardulyn, and Smits 2017). We repeated the assemblies using different K‐mer sizes (i.e., 22, 33, 60, and 100), and the cytochrome c oxidase subunit 1 (*cox1*; 1551 bp) and NADH dehydrogenase subunit 1 (*nd1*; 975 bp) as seed sequences, both obtained from the published mitogenome (ON260847.1), to improve the recovery of mitochondrial genomes. Mitochondrial genomes were annotated using MITOS2 (Bernt et al. 2013) using default parameters. Geneious Prime 2023.2.1 (https://www.geneious.com) software was subsequently used for sequence and annotation validation, MUSCLE v‐5 (Edgar 2022) for alignment and the extraction of sequences from specific genes. FASCONCAT v‐1.11 (Kück and Meusemann 2010) was used for gene concatenation. We transformed the mitochondrial dataset into nexus format with DnaSP v‐61,203 (Rozas et al. 2017) and constructed the haplotype network using a Median‐Joining (MJ) approach with PopART v‐1.7 (Leigh and Bryant 2015). Finally, the *F*_ST_ between spring and autumn spawner mitogenomes was calculated in Arlequin v.3.5.2.2 (Excoffier and Lischer 2010) with 1000 permutations.

## 3 Results

### 3.1 Spring–Autumn Spawners Phenotypic Analyses

**FIGURE 2:**
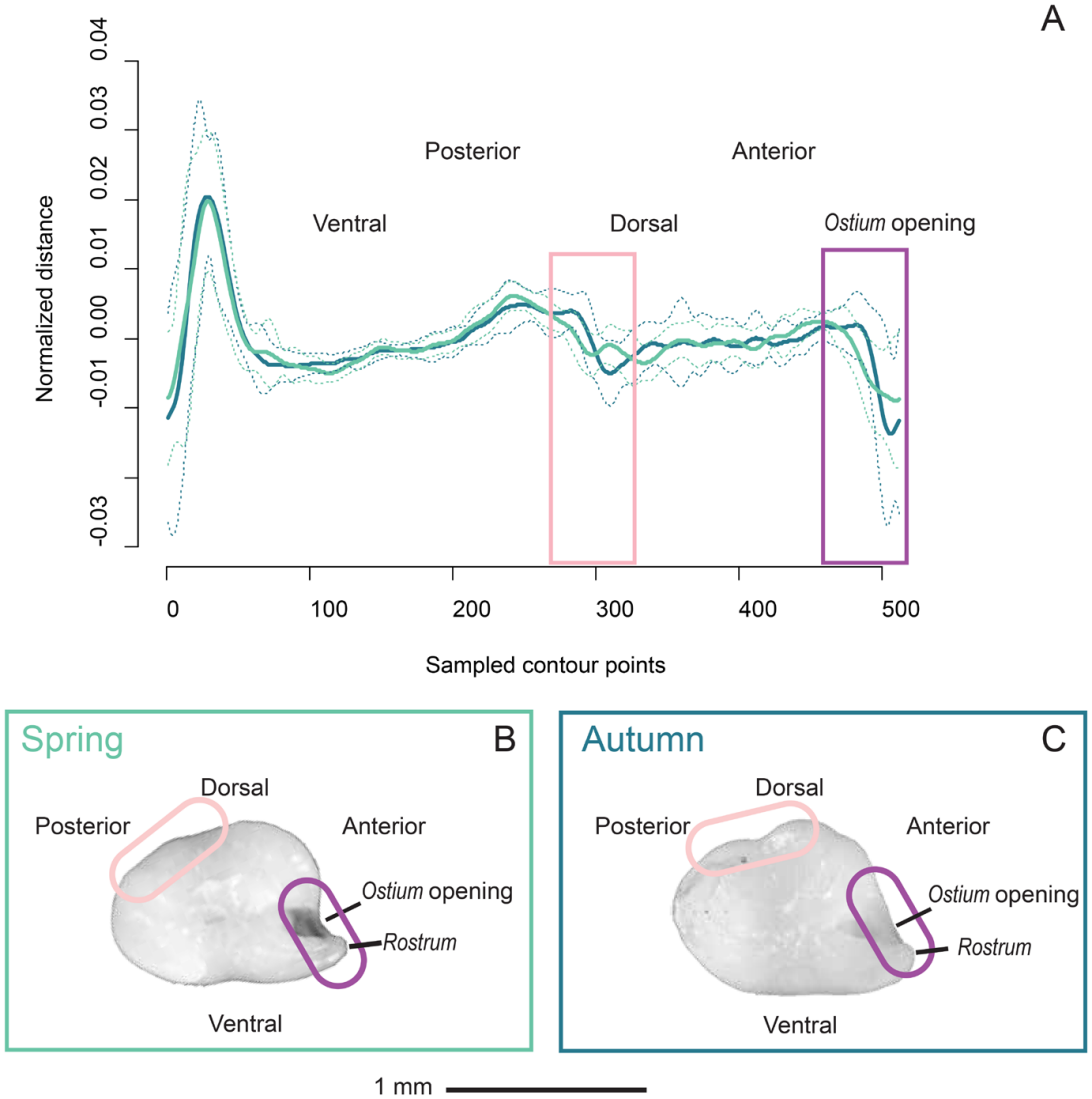
Spring and autumn otoliths analysis. (A) Average decomposition of otolith contour (i.e., Cartesian coordinates) using the 4th wavelet for spring (light green) and autumn (dark blue) spawners starting from the *rostrum* and moving clockwise, together with the standard variation of the respective average. *X*‐axis represents the 512 equidistant points of contour and *Y*‐axis the mean normalized distance from the centroid of the otolith to the edge. Otolith picture of a (A) spring spawner and (C) autumn spawner. The areas differentiating the two groups of spawners by PC7 (light pink) and PC4 (purple) are marked in the decomposition of otolith contour and otolith pictures.

Age estimations based on individual otolith information revealed that both spring and autumn spawners were composed of 2‐ and 3‐year‐old individuals (Table S1). The two‐sample Kolmogorov–Smirnov test confirmed no significant differences in age distribution between the two spawning groups in both localities (SOR: *p* = 1, NAM: *p* = 0.74). Similarly, no significant differences in size (total length) were found between spring and autumn spawners in none of the two localities (SOR: *p* = 0.84, NAM: *p* = 0.09). Morphological differences in otolith shape were apparent between spring and autumn spawners. The performance of the model used, evaluated with the confusion matrix (see methods), revealed an overall accuracy of 95.12%, with a 95% confidence interval ranging from 87% to 100%. The Kappa statistic computed from the confusion matrix was 0.902, reinforcing the model's capability to classify seasons with a substantial level of agreement. The variable importance analysis revealed that, among all principal components, PC7 and PC4 were the most significant predictors of the spawning season, showing significant differences between the two seasons (Figure 2, Figure S1). The PC7 component captured the morphological variability between the posterior and dorsal margins, being entire in spring spawners and slightly lobed in autumn ones (Figure 2). The PC4 component explained differences in the development of the antero‐dorsal part of the otolith, particularly influencing the size of the ostium opening and *rostrum* with a generally shorter *rostrum* in autumn spawners compared to spring spawners (Figure 2).

### 3.2 Genetic Diversity and Divergence Highlight Chromosomal Regions of Interest

Following quality filtering of raw sequence data, the average yield was 53 [±11] million PE reads per sample. All reads were successfully aligned to the reference genome, with an average coverage of 14.7× per individual. Genotyping across the entire cohort identified 1,939,545 SNPs distributed over all 25 chromosomes, with less than 5% missing data per individual (Table S2). No significant differences between spring and autumn spawners were found for nucleotide diversity (*π*) (*t*‐test = −0.1, *p*‐values: 0.9) with an average of 33.5 ± 27.8, nor for the observed heterozygosity (*H*_o_) (*t*‐test = −0.92, *p*‐values: 0.36).

The results from AMOVA revealed distinct patterns of genetic differentiation. When localities were considered as the primary hierarchical level, no significant differences were observed between localities (*p*‐value: 0.58). However, significant differences were detected between seasons within localities (*p*‐value: 0.01) and within individual samples (*p*‐value: 0.01). Of the total variance, 99% was attributed to differences within samples, while 0.85% was due to seasonal differences within localities. Conversely, when seasons were treated as the primary hierarchical level, no significant differences were found between seasons (*p*‐value: 0.19). Yet, significant differences emerged between localities within seasons (*p*‐value: 0.01) and within individual samples (*p*‐value: 0.01). Again, 99% of the total variance was explained by differences within samples, but only 0.33% was accounted for by differences between localities within seasons. Thus, the variance explained by seasonal differences within localities was greater than that explained by locality differences within seasons.

To evaluate the impact of library preparation in genetic differentiation, we plotted the first three components of the multidimensional scaling (MDS) analysis. The second component segregates the individuals based on their library preparation protocols (i.e., low input DNA library or automatized (normal) library; Figure S2). Interestingly, samples prepared with the low input DNA library presented significantly different characteristics as assessed with the Wilcoxon test than the normal libraries prepared with the automatized protocol (Figure S2). Interestingly, the low input libraries presented a significantly higher number of reads (*W* = 612, *p*‐value: 3.0e‐06), higher coverage (*W* = 612, *p*‐value: 3.0e‐06), higher observed heterozygosity (*W* = 634, *p*‐value: 4.7e‐07), and lower missing data (*W* = 40, *p*‐value: 1.2e‐06). Thus, the second component of the MDS indicated slightly higher heterozygosity values arising from higher number of reads per sample, and thus only the first and third components were used for further analyses.

**FIGURE 3:**
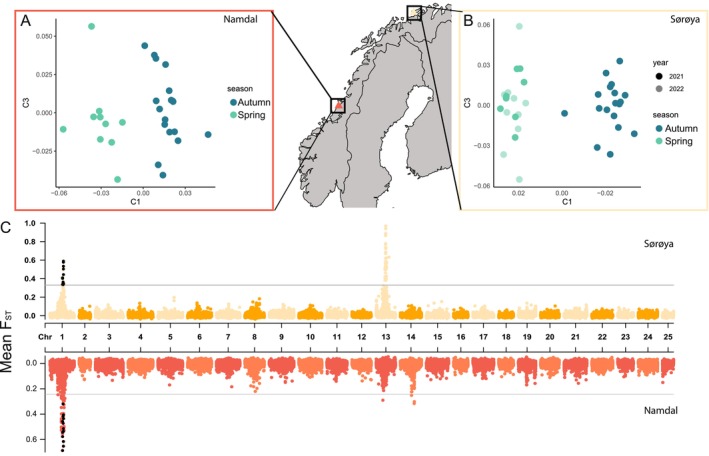
Genetic divergence between spring and autumn spawners. MDS plots showing the genetic clustering of spring and autumn spawners in (A) Namdal and (B) Sørøya. (C) Manhattan plots of *F*_ST_ values between spring and autumn spawners across the 25 chromosomes for Sørøya (yellow) and Namdal (red). Black dots highlight non‐overlapping windows (10,000 bp) shared between both localities (significant according to Rosner test) exceeding the 99.9th percentile (gray horizontal line) of the empirical *F*_ST_ distribution.

When we identified the two groups of spawners and analyzed each locality separately, the first MDS component revealed strong independent clustering of spring and autumn spawners in both sampled localities (Figure 3A), as suggested by the AMOVA group test. Moreover, SOR samples collected in spring 2021 and spring 2022 (Figure 3A) did not show interannual differences and clustered together both with the first and third MDS components. Therefore, 2021 and 2022 SOR spring samples were combined for subsequent analyses.

The genome‐wide *F*_ST_ calculation based on 10,000 bp non‐overlapping windows between spring and autumn spawners revealed high genetic divergences in specific genomic regions. Regions of chromosome 1 presented windows exceeding the 99.9th percentile of the empirical distribution of *F*_ST_ values. These regions also showed a high absolute allele frequency difference when comparing the spring and autumn spawners (Figure S3). A total of 14 regions, marked as black dots, were shared between both localities (Figure 3B), all of them significant according to the Rosner test. Other regions of high differentiation between spring and autumn spawners, both with *F*_ST_ values and absolute allele frequency differences, were found in chromosome 13 for SOR and chromosomes 13 and 14 in NAM (Figure 3, Figure S4). However, none of those chromosome regions were identified in both localities as differentiating the two spawning groups.

The zoom in on chromosome 1 revealed that the genetic divergence between spring and autumn spawners in each locality on this chromosome is concentrated in specific regions, with most of the chromosome showing low levels of divergence (Figure S3). A total of 281 SNPs on chromosome 1 were significantly different between both seasons according to the Rosner test and shared between both localities (Figure S3A). Those SNPs also showed high absolute allele frequency difference when comparing the spring and autumn spawners in both localities (Figure S3B). We did not find signals of inversion separating the two spawning groups on the chromosome 1 (Figure S5).

### 3.3 Functional Analysis of Genomic Regions Reveals Association With Key Biological Processes

**FIGURE 4:**
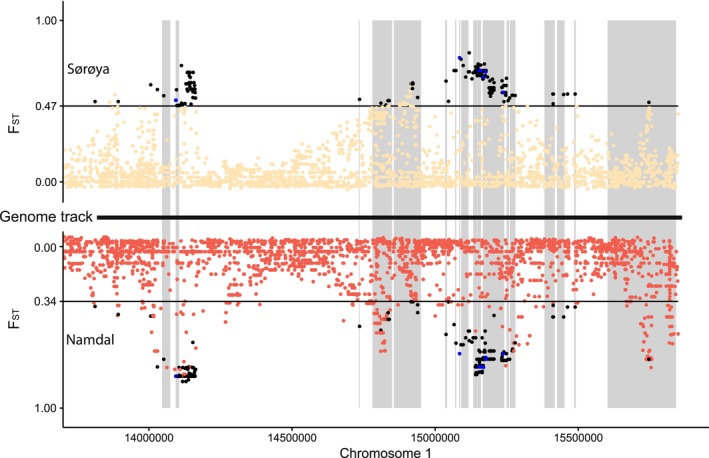
Detailed view of chromosome 1 highlighting regions of high genetic divergence. Manhattan plot zooming in on the divergent part of chromosome 1, comparing *F*_ST_ SNP values between spring and autumn spawners in Sørøya (yellow) and in Namdal (red). The gray boxes depict the location of the genes associated with the shared outlier SNPs (Table S4). The black and blue dots represent the 281 significant SNPs shared between both localities. The eight blue dots correspond to outlier SNPs changing amino acid (nsSNPs).

Among the 281 SNPs significantly different between both spawning groups and shared between both locations, 184 SNPs were distributed within 18 genes (Figure 4). These 18 genes predominantly play roles in regulation and inflammatory responses (Tables S1 and S4). Among the 18 genes where putative outliers were found, seven showed SNPs in their exons, five in their untranslated regions (UTR) and 14 in the intron regions (Table S3). The highest *F*_ST_ values were found in cabp4 and doc2d genes. Both genes share common functions related to the regulation of neurotransmitter release, particularly in the context of calcium ion‐dependent processes (Table S3). However, all SNPs for cabp4 doc2d were found in intronic regions. Of the 19 SNPs found in exons, 8 resulted in non‐synonymous (nsSNPs) changes (Figure 4, Table S5) and were distributed in four different genes (card14, serping1, lonrf1l, and dlc1). The eight nsSNPs showed contrasting allele frequencies and significant genotype frequency differences between spring and autumn spawners and were at Hardy–Weinberg equilibrium in each season, combining individuals of the two locations (Table S5). The eight nsSNPs result in amino acid changes that alter their properties, affecting classifications based on R‐group characteristics, nutritional value, and metabolic fate (Table S5).

### 3.4 Mitochondrial Analysis

The complexity of the lumpfish's mitochondrial genome (17,266 bp), characterized by GC‐rich regions (Maduna et al. 2022), presented challenges in our assembly analyses. A total of 28 mitogenomes circularized successfully with an assembly size ranging from 17,260 to 17,580 bp. For the non‐circularized mitogenomes, the assembly size varied between 14,338 and 19,338 bp. Even though circularization was not achieved in most of the samples, the average mitogenome coverage per individual ranged from 754× to 85,049×. Among the non‐circularized mitogenomes, eight individuals had deletions within the *nad1* gene. Consequently, to ensure comparisons only among orthologous regions and the absence of missing data, we concatenated the gene sequences which were present in all samples (the partial region of the *nad1* was also included), resulting in a total sequence alignment of 14,048 bp for the 64 individuals comprising 13 protein coding and 2 rRNA genes.

The mitochondrial haplotype network, constructed from the concatenated mitogenome dataset, showed a radiating haplogroup found in both spring and autumn spawners (Figure S6) and a few haplotypes which were separated by multiple mutations (from 1 to 59). *F*_ST_ analyses statistically supported this observation, since no significant mitogenome differentiation was found between individuals that spawn in spring and those that spawn in autumn (*F*_ST_ = 0.027; *p*‐value = 0.132). Interestingly, three individuals (S22S15, N21F25, and N22S13) representing different spawning times and locations exhibited high genetic divergence. These individuals did not show a reduced number of reads, neither large number of missing loci (Table S2) nor a reduced coverage at the mitogenome (75,257×, 7558×, and 32,173×, respectively), and we can therefore be confident that the observed genetic divergence is attributed to true nucleotide changes.

## 4 Discussion

Our study on Atlantic lumpfish reveals significant genetic and phenotypic divergence between spring and autumn spawners, underscoring the pivotal role of allochrony in shaping the genetic structure of marine species. Our findings unveil previously undetected genetic groups, with adaptations to the distinct seasonal environmental conditions faced by spring and autumn spawners, that should be considered in management of this commercially exploited species, and for understanding adaptation and evolution in marine organisms.

### 4.1 Genetic Divergence and Temporal Segregation

In our study, we identified a pronounced genetic structure that differentiates spring from autumn female spawners of Atlantic lumpfish. We observed differential genetic divergence across the genome suggesting the presence of putative genomic island of divergence (Nosil and Feder 2012). On chromosome 1, we observed the same genetic divergence between the two spawning groups in the two localities, suggesting parallel evolutionary responses (Torrado et al. 2022). Such patterns imply that this chromosomal segment likely harbors genes under high selective pressure, which may be related to environmental mechanisms driving reproductive isolation (Wolf and Ellegren 2017). The emergence of reproductive isolation and/or adaptive divergence is facilitated by the existence of chromosomal inversions (Berdan et al. 2023). However, in the present work, we did not find signals of inversion separating the two spawning groups. Similar patterns of genetic differentiation have been observed in Chinook salmon (*Oncorhynchus tshawytscha)*, where differentiation between early and late runners was linked to a specific region on chromosome 28 and without chromosomal inversions being reported (Thompson et al. 2020). Moreover, genomic islands of divergence despite gene flow have been found not related to previously identified inversions. This suggests that other evolutionary forces may allow these regions to evolve independently (Weist et al. 2022). These regions can facilitate adaptation to different environmental or temporal conditions, thus being naturally selected, and maintained by allochrony. Our study also revealed regions of genetic divergence between spring and autumn spawners in chromosomes 13 and 14. However, these regions were not shared between both localities, indicating potential adaptations to specific environmental conditions in each locality (Sodeland et al. 2016). Further studies across different localities considering these two spawning periods and for different years will be able to tackle whether these regions are relevant for local adaptation and their interaction with more complex allochronic processes.

### 4.2 Functional Analysis and Environmental Adaptation in Atlantic Lumpfish

The functional analysis of the genomic islands of divergence present on chromosome 1 revealed 18 genes predominantly associated with the regulation of molecular functions, including responses to stimuli, developmental processes, and various aspects of the inflammatory response, such as responses to oxidative stress, nitrogen compounds, nitric oxide, and general stress. This genetic variation suggests that the two spawning groups of Atlantic lumpfish may have adapted to distinct environmental stressors or different physiological needs for spawning and/or larval development. This genetic divergence aligns with the marked seasonal differences in biotic and abiotic conditions along the Norwegian coast (Ibrahim et al. 2014). For instance, coastal temperatures peak after spring bloom, coinciding with a decrease in nitrate concentration during the spring bloom, while winter features more mixed layers with lower temperatures and higher nitrate concentrations. The identified genes likely play a crucial role in enabling lumpfish to cope with these environmental variations.

Among those 18 genes, four genes presented nsSNPs which strongly differentiate spring and autumn spawners. Amino acid substitutions can significantly impact protein folding and therefore their function and metabolism. This can also confer resistance to toxic dietary compounds that can lead to evolutionary adaptations, enhancing protein functionality in new environments (Pegueroles et al. 2016). Changes in the R group can affect protein folding and stability, potentially disrupting essential interactions like hydrophobic bonds altering the protein's surface properties, influencing interactions with other molecules and affecting solubility and binding characteristics (Lopez and Mohiuddin 2021). The transition from essential to non‐essential amino acids, as seen in the present study in the Leu/Pro substitution in the *dlc1* gene, indicates a potential adaptation to synthesize these amino acids endogenously under specific conditions (Lopez and Mohiuddin 2021), possibly related to the metabolic demands during different spawning periods. Finally, the change in metabolic fate from ketogenic to glucogenic amino acids could influence energy metabolism pathways (Wei et al. 2021; Xu et al. 2022), which are critical during energy‐intensive periods such as spawning. Proper implications of a change of amino acid are too complex to be explained by the present data; further analysis is required to conclusively determine the impact of these changes.

The presence of nsSNPs has also been observed in a specific region of strong genetic divergence in the Chinook salmon when comparing early and late runners (Thompson et al. 2020). Those nsSNPs were found within *GREB1L* coding protein gene, which also exhibited relative frequences highly associated with the running time. *GREB1L* is a central regulator of vertebrate development, specifically affecting renal, gonadal, and inner ear organ systems (Brophy et al. 2017; Schrauwen et al. 2018). The inner ear of fish is a sensory organ that plays a crucial role in maintaining balance and hearing, primarily through the formation and function of otoliths, calcified structures that assist in these sensory processes. Interestingly, in our study, even if all SNPs in *cabp4* and *doc2d* were found in intronic regions, those genes showing the highest *F*_ST_ values in both localities are known to be involved in calcium signaling, which could affect metabolic structures such as otoliths. Otoliths, primarily composed of calcium carbonate, rely on calcium transport mechanisms for their growth and maintenance (Cruz et al. 2009; Groffen et al. 2006). In the present study, we found differences in the otolith shape between spring and autumn spawners. Specifically, the autumn spawners exhibit a less pronounced *rostrum*, but an expanded *ostium* area—the site where sensory tissue comes into contact with the otolith (Aguirre 2003). An increase of *ostium* has been associated to adaptations to low‐light or turbid water conditions (Aguirre and Lombarte 1999; Lombarte and Lleonart 1993; Verocai et al. 2023). The association between specific loci and ecotypes (or phenotypes) related to otolith morphology has been observed in other species, such as *Gadus morhua* (Cardinale et al. 2004; Jonsson et al. 2021) or *Lutjanus kasmira* (Vignon and Morat 2010), where genetic variations correlate with adaptations to different environmental conditions. This suggests that the divergence in otolith shape between the two spawning groups of lumpfish may reflect a form of phenotypic adaptation, potentially underpinned by genetic factors that confer an adaptive advantage in response to the seasonal variations. Incorporating environmental data and including additional samples across years would help clarify the role of environmental variables in the observed genetic structure and otolith shape divergence between spring and autumn lumpfish spawners.

### 4.3 Implications for Gene Flow and Genetic Structure

The presence of the genomic islands of divergence, alongside the general lack of differentiation across most of the genome including the mitochondrial genome, suggests that the observed divergence between spring and autumn spawners might be a relatively recent phenomena where rapid selection processes have not yet allowed widespread genomic divergence (Sendell‐Price et al. 2020). Alternatively, ongoing gene flow between the spawning groups could slow the accumulation of divergence by exerting a homogenizing effect on loci that are neutrally evolving or under weak selection (Nosil et al. 2017). A plausible explanation for ongoing gene flow could be overlapping spawning periods between the groups. Assortative mating often carries a cost (Gavrilets 2004, 2005), suggesting that when both populations are present during a shared spawning window, the evolution of assortative mating could be hindered (Taylor and Friesen 2017). Although spring and autumn are believed to represent the main peaks of spawning, lumpfish may spawn throughout the year (pers. comm. Trude C. Halvorsen, Sørøya Rensefisk AS). Future research should therefore adopt a comprehensive temporal sampling scheme to identify potentially admixed individuals.

### 4.4 Sampling Strategies and Management Implications

The elucidation of the temporal genetic structure in Atlantic lumpfish has implications that transcend the species itself. Indeed, our research underscores the importance of integrating temporal factors into genetic study designs. Without careful consideration of the timing of sample collection, particularly in relation to spawning events, there is a risk of conflating genetically distinct cohorts, leading to erroneous interpretations of population structure. For instance, in Atlantic lumpfish, Jansson et al. (2023) observed that samples comprising solely spawning individuals or offsprings formed well‐defined regional clusters. In contrast, samples from immature migrating fish, offshore captures, and those containing a mix of juveniles and adults exhibited greater genetic admixture. Indeed, the presence of a highly admixed East Atlantic group in their study raises questions about its composition. It is uncertain whether this group represents a genuinely panmictic population, as previously proposed (Jónsdóttir et al. 2018, 2022; Whittaker, Consuegra, and Garcia de Leaniz 2018). It could be an artifact of sampling individuals from different spawning cohorts outside their spawning season, thus blending genetic material from both spring and autumn spawners. Similarly, Langille et al. (2023) collected samples from individuals without specific regard to maturity, thereby amalgamating data from adults at various stages of maturity with juveniles. Their analysis of the genetic structure among the northernmost individuals identified two distinct clusters: one consisting exclusively of adult individuals and another comprising all juveniles and some adults, collected from the same area as the first group. The discrete grouping observed could not be conclusively attributed to relatedness or sex‐biased sampling and lead the authors to hypothesize a cryptic coastal life history form in the northern part of their study area in the Northwest Atlantic (Langille et al. 2023). However, the genetic structure observed could be influenced by the collection of individuals away from their spawning grounds and at different stages of maturity, potentially confounding the interpretation of the data. Some species may show philopatric breeding behavior but undergo long feeding migrations forming mixed stock groups (Carreras et al. 2011; Clusa et al. 2016). These studies exemplify the complexities and potential missteps of non‐specific sampling approaches. They emphasize the necessity for meticulously planned sampling strategies that account for the life history traits and reproductive behaviors of the target species.

For management purposes, understanding that the species exhibits temporal genetic structure is crucial. Identifying specific genomic regions associated with spawning times could aid in the development of genetic markers for monitoring population structure and dynamics, which is essential for the management of fisheries and the conservation of biodiversity. This is particularly relevant for fisheries focusing on harvesting roe from Atlantic lumpfish for human consumption, which predominantly target near‐spawning individuals during the spring season. Consequently, only the spring spawning component faces fishing mortality, highlighting the need for careful management of this specific group to prevent overexploitation. Additionally, for lumpfish producers using these species as cleaner fish in salmon farms, our findings are significant. Artificially breeding individuals from different spawning times could result in poor‐quality offspring due to potential mismatches in optimal spawning periods or other life history traits. Notably, malformations in offspring from crosses between spring spawning males and autumn spawning females have been observed by Trude Caroline Halvorsen from Sørøya Rensefisk AS (personal communication). Although anecdotal, this raises important questions about the genetic and environmental factors influencing broodstock performance and the evolutionary consequences in breeding these two groups. It could suggest that segregating broodstock by their natural spawning times, acknowledging the genetic differences between spring and fall spawners, could be crucial for optimizing production outcomes. This point could be particularly relevant for aquaculture practices, as it underscores the need for further investigation into the implications of hybridization between closely related groups potentially reproductively isolated by breeding in different seasons.

In conclusion, this research significantly contributes to our comprehension of allochrony within evolutionary biology and ecology, offering vital insights that can be leveraged to improve conservation strategies and management practices. By integrating these findings, we can better ensure the sustainable use and preservation of marine resources, ultimately supporting the health and resilience of marine ecosystems.

## Author Contributions

**Mathilde Horaud:** conceptualization (lead), data curation (equal), formal analysis (equal), resources (equal), visualization (lead), writing – original draft (lead), writing – review and editing (lead). **Núria Raventós:** conceptualization (equal), formal analysis (equal), methodology (lead), resources (equal), writing – review and editing (equal). **Kim Præbel:** conceptualization (equal), funding acquisition (equal), resources (equal), writing – review and editing (equal). **Carles Galià‐Camps:** formal analysis (equal), methodology (equal), writing – review and editing (equal). **Cinta Pegueroles:** formal analysis (equal), methodology (equal), resources (equal), writing – review and editing (equal). **Carlos Carreras:** formal analysis (equal), methodology (equal), resources (equal), writing – review and editing (equal). **Marta Pascual:** formal analysis (equal), methodology (equal), resources (equal), writing – review and editing (equal). **Victor M. Tuset:** formal analysis (equal), methodology (equal), writing – review and editing (equal). **Shripathi Bhat:** data curation (equal), methodology (equal), resources (equal), writing – review and editing (equal). **Arve Lynghammar:** conceptualization (equal), writing – review and editing (equal).

## Conflicts of Interest

The authors declare no conflicts of interest.

## Benefit‐Sharing Statement

This study complies with the principles of the Convention on Biological Diversity and the Nagoya Protocol. The fish specimens utilized in this research were obtained from Norwegian lumpfish producers during routine operations. Leftover carcasses were used for this study, thereby avoiding the need for active collection of live specimens and negating the requirement for ethical approval. All researchers involved in this study are acknowledged as co‐authors, ensuring fair recognition of their intellectual contributions.

## Supporting information


Appendix S1.

## Data Availability

The raw short‐read sequences utilized for all bioinformatics analyses in this study have been deposited in the National Center for Biotechnology Information Sequence Read Archive under BioProject ID: PRJNA1218516. The SNP genotype data and otolith 4th wavelet data used in this study are deposited in *DataverseNO* (Horaud et al. 2025).
