# Supplementary material for: Allochrony in Atlantic Lumpfish: Genomic and Otolith Shape Divergence Between Spring and Autumn Spawners
Source: Ecol Evol. 2025 Feb 14;15(2):e70946. doi: 10.1002/ece3.70946 (PMC11826085; doi:10.1002/ece3.70946)
Supplement: Supplementary file 1 — Appendix S1. [file ECE3-15-e70946-s001.zip › ece370946-sup-0001-AppendixS1.docx]

# Supplementary


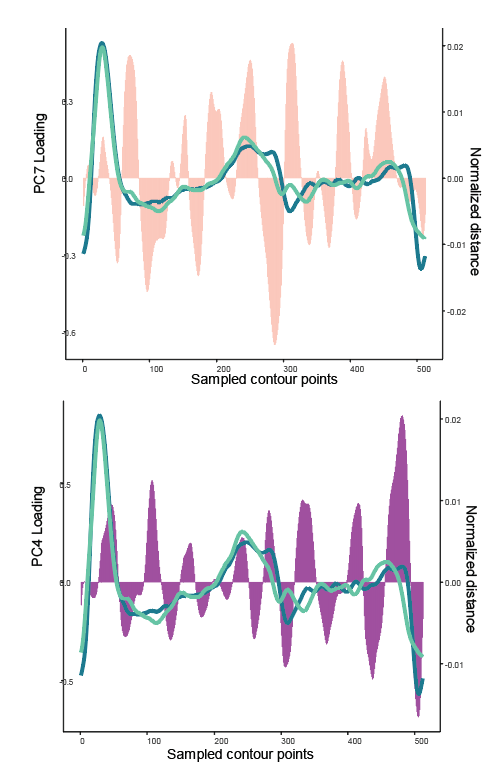


**Figure S1**: Loading plots of the principal components PC7 (light pink) and PC4 (purple) that explain the most the differences in shape of the sagitta between spring and autumn spawners. The loadings are plotted against the Average decomposition of otolith contour using the 4^th^ wavelet for spring (light green) and autumn (dark blue) spawners.


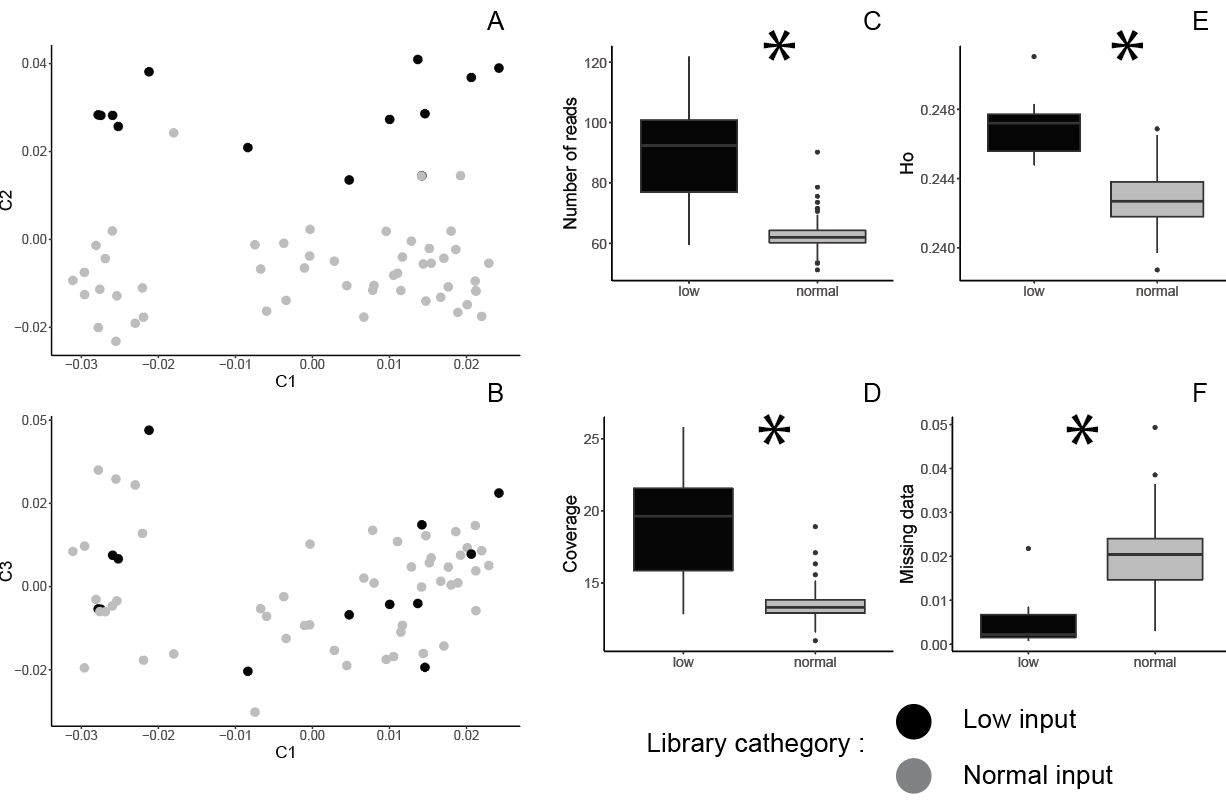


**Figure S2:** MDS of spring and autumn spawners in SOR and NAM using A) the first (C1) and second (C2) components and B) the first and third (C3) components. The second component seems to slightly distribute the data according to their library preparation protocol. Boxplots showing mean values between low and normal input library for C) number of raw reads, D) coverage, E) observed heterozygosity and F) missing data per individual. Asterisks indicate significant differences between low and normal input library according to the Wilcoxon-test.


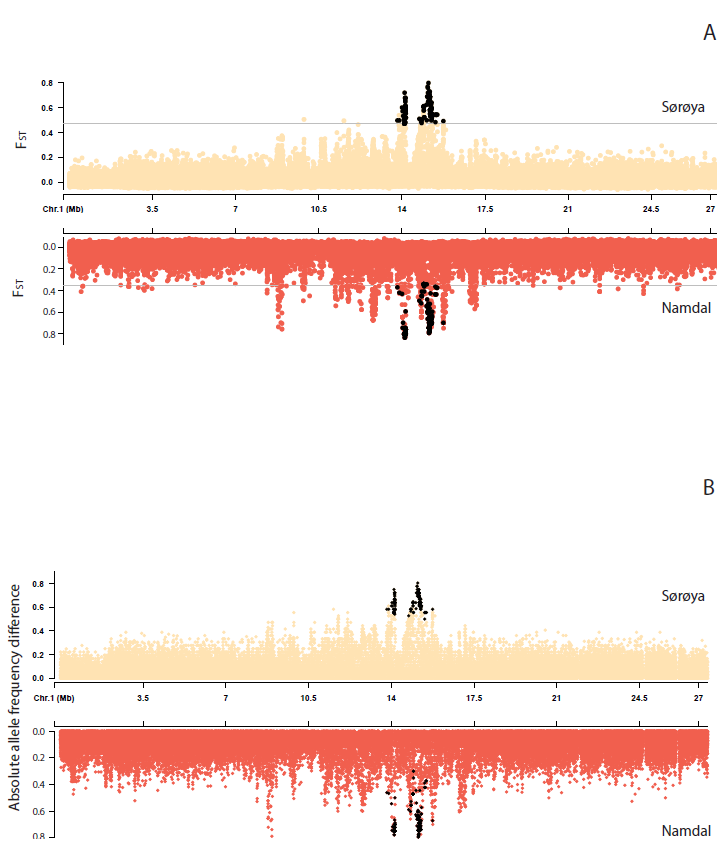


**Figure S3:** Manhattan plots zooming in on chromosome 1 comparing spring and autumn spawners in Sørøya (SOR) (yellow) and in Namdal (NAM) (red) on a SNP basis, using A) F_ST_ values, and B) the absolute allele frequency differences per SNP. The black dots **indicate** the position of the 281 SNPs shared between both localities exceeding the 99.9^th^ percentile of the empirical distribution of F_ST_ and significant according to Rosner test.


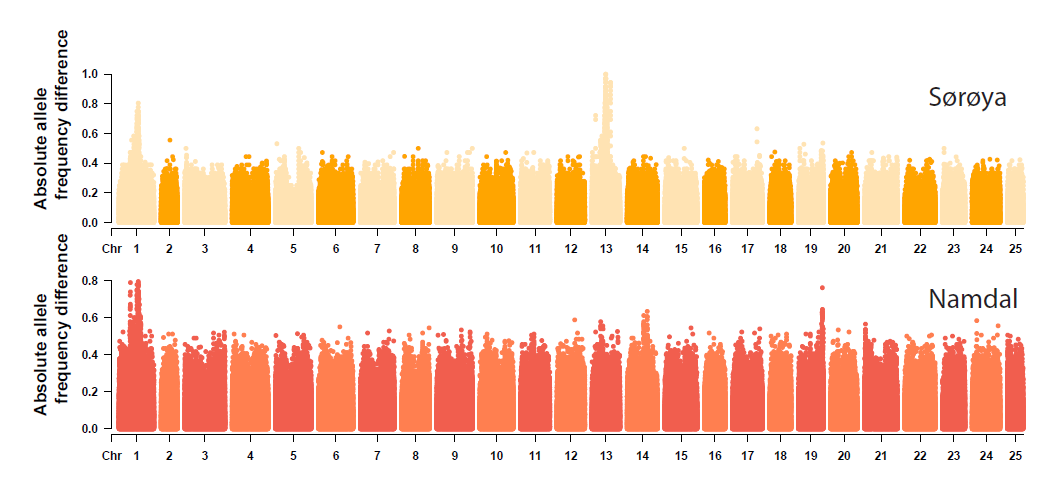


**Figure S4:** Manhattan plots of the absolute allele frequency difference per SNP between spring and autumn spawners across the 25 chromosomes in Sørøya (up) and Namdal (down)


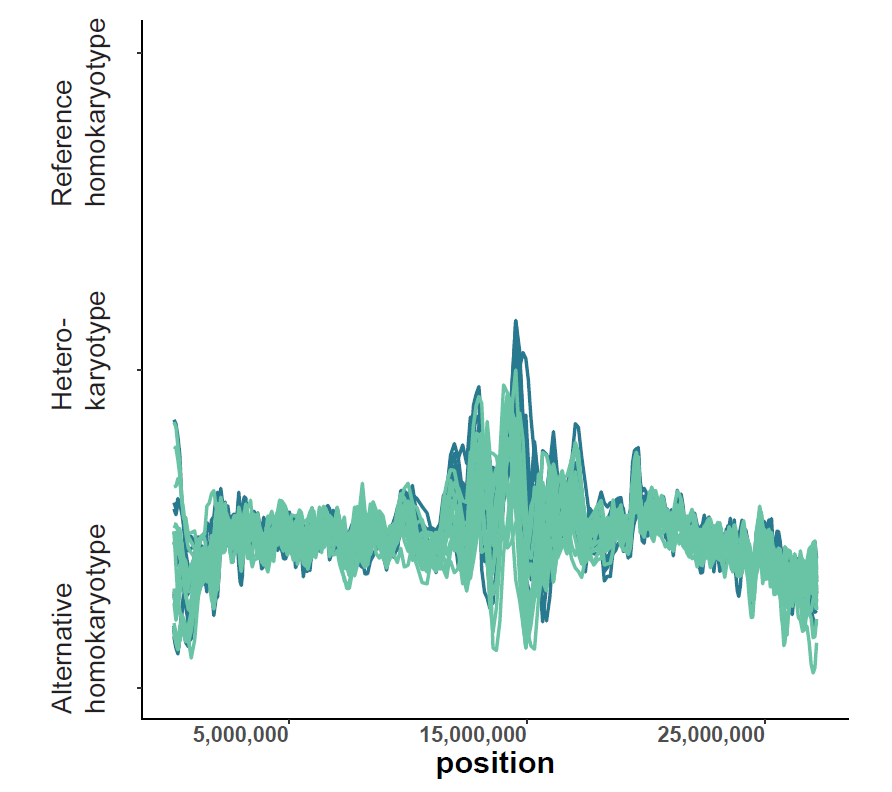


**Figure S5:** Plots of genotypes along the chromosome 1 to check for inversions. For each spring (green) and autumn (bleu) spawners we plotted their average genotype on a 1,000 bp window, with 250 bp sliding windows.


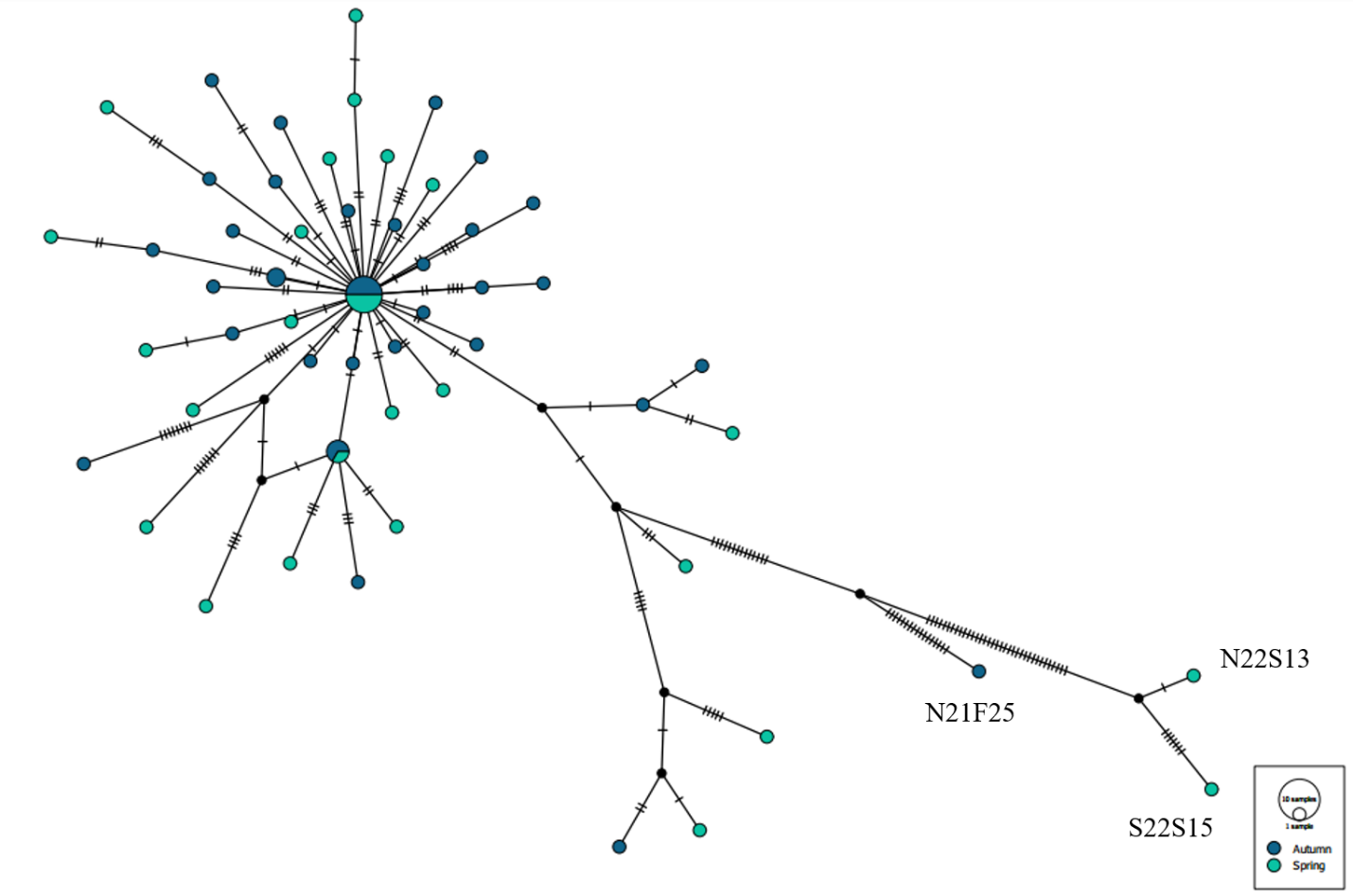


**Figure S6**: **Haplotype network of spring and autumn spawners mitogenomes**. Each mitogenome contains the concatenated dataset of the 13 protein coding genes and the 2 rRNA genes. Each circle represents a unique mitochondrial haplotype, with the circle size proportional to the frequency of the haplotype color coded according to spawning season. The number of hatch marks on each line connecting haplotypes denotes the number of changes needed to connect the different haplotypes. Small, black circles along the lines indicate intermediate unsampled haplotypes. Note that line length is not proportional to the number of substitutions between linked haplotypes.

**Table S1**: Information of each analyzed mature female. Individual identifier (ID), location, year and season of sampling. Individual total length (TL), sagittal otolith size (TOT_SZ) and age estimation in years based on the otoliths banding pattern (Age).

**Table S2**: Sequencing information per individual. Individual identifier (ID), location, year and season of sampling. Library type (low or normal DNA input), number of raw reads merged, and number of cleaned reads after trimmomatic. Percentage of mapped reads, mean SNP coverage and frequency of PCR duplicates in the raw bam files. Mean SNP coverage after duplicates removal, percentage of missing data, and observed heterozygosity for each individual in the final vcf file. Note that the number of polymorphic markers in the final dataset was of 1,939,545 SNPs.

**Table S3:** Genes on chromosome 1 with the same outlier SNPs in both localities. Each row represents a different gene, providing the gene name, the start and end positions based on the genome annotation, as well as their functions when available. We also provide the number of SNPs in exons, in introns and in 5’ and 3’ UTR regions. For SNPs in exons, we indicate whether they produce synonymous (S) or non-synonymous (NS) changes.

**Table S4:** Gene Ontology (GO) terms that have been processed through Revigo to minimize redundancy and highlight the most representative terms within specific biological categories for the 18 genes in Table S3. TermID: The unique identifier for each Gene Ontology (GO) term. Name: The descriptive name of the GO term, which provides insight into the biological function, process, or component represented. LogSize: Represents the logarithm of the cluster size to which the GO term belongs, indicating the relative size of the group of related terms. This helps in understanding the breadth of the biological theme covered by the cluster. Frequency: The frequency of the GO term within the dataset, showing how often the term is associated with the genes under study. This can indicate the prevalence of certain biological processes in the dataset. Uniqueness: A measure of how unique the GO term's role is within its cluster, with higher values indicating more unique contributions to the biological processes. This helps in identifying terms that provide distinct insights into the biological mechanisms. Dispensability: A metric indicating how essential the GO term is within its cluster; lower values suggest that the term is less dispensable and more critical. This is crucial for understanding which terms are fundamental to the biological themes being studied. Representative: Indicates whether the GO term is considered a key representative in its cluster. If a term is a representative, it is marked with a specific cluster number (e.g., 302); if not, it is marked as 'null'. This column highlights the terms that are central to summarizing the cluster's theme.

**Table S5: Information of the** **nsSNPs.** Columns include SNP position in the genome, gene name, reference and alternative SNP alleles (REF/ALT) according to the reference genome, amino acid changes (aa REF/ALT), and classifications of amino acids based on their R-group (basic, acidic, polar and non-polar), nutritional value (E:essential and NE:non-essential), and metabolic fate (G:glucogenic and K:ketogenic). The observed genotypes for spring and autumn spawners combining the two locations (0=reference allele, 1=alternative allele). Genic frequencies of the reference allele for spring and autumn spawners. Chi-square values comparing observed and expected genotype frequencies to test for Hardy-Weinberg equilibrium in Spring and Autumn spawners (values < 3.84 do not significantly differ from equilibrium). Chi-square values testing for seasonal differences on genotype frequency distribution (values > 5.99 are significantly different).
